# Breastmilk-Saliva Interactions Boost Innate Immunity by Regulating the Oral Microbiome in Early Infancy

**DOI:** 10.1371/journal.pone.0135047

**Published:** 2015-09-01

**Authors:** Saad S. Al-Shehri, Christine L. Knox, Helen G. Liley, David M. Cowley, John R. Wright, Michael G. Henman, Amitha K. Hewavitharana, Bruce G. Charles, Paul N. Shaw, Emma L. Sweeney, John A. Duley

**Affiliations:** 1 School of Pharmacy, The University of Queensland, Brisbane, Australia; 2 College of Applied Medical Science, Taif University, Taif, Saudi Arabia; 3 Institute of Health and Biomedical Innovation, Faculty of Health, Queensland University of Technology, Brisbane, Australia; 4 Mater Research Institute, Mater Health Services, Brisbane, Australia; 5 School of Veterinary Science, The University of Queensland, Gatton, Australia; Instituto de Biociencias—Universidade de São Paulo, BRAZIL

## Abstract

**Introduction:**

Xanthine oxidase (XO) is distributed in mammals largely in the liver and small intestine, but also is highly active in milk where it generates hydrogen peroxide (H_2_O_2_). Adult human saliva is low in hypoxanthine and xanthine, the substrates of XO, and high in the lactoperoxidase substrate thiocyanate, but saliva of neonates has not been examined.

**Results:**

Median concentrations of hypoxanthine and xanthine in neonatal saliva (27 and 19 μM respectively) were ten-fold higher than in adult saliva (2.1 and 1.7 μM). Fresh breastmilk contained 27.3±12.2 μM H_2_O_2_ but mixing baby saliva with breastmilk additionally generated >40 μM H_2_O_2_, sufficient to inhibit growth of the opportunistic pathogens *Staphylococcus aureus* and *Salmonella spp*. Oral peroxidase activity in neonatal saliva was variable but low (median 7 U/L, range 2–449) compared to adults (620 U/L, 48–1348), while peroxidase substrate thiocyanate in neonatal saliva was surprisingly high. Baby but not adult saliva also contained nucleosides and nucleobases that encouraged growth of the commensal bacteria *Lactobacillus*, but inhibited opportunistic pathogens; these nucleosides/bases may also promote growth of immature gut cells. Transition from neonatal to adult saliva pattern occurred during the weaning period. A survey of saliva from domesticated mammals revealed wide variation in nucleoside/base patterns.

**Discussion and Conclusion:**

During breast-feeding, baby saliva reacts with breastmilk to produce reactive oxygen species, while simultaneously providing growth-promoting nucleotide precursors. Milk thus plays more than a simply nutritional role in mammals, interacting with infant saliva to produce a potent combination of stimulatory and inhibitory metabolites that regulate early oral–and hence gut–microbiota. Consequently, milk-saliva mixing appears to represent unique biochemical synergism which boosts early innate immunity.

## Introduction

Nucleotides play an essential role in many cellular processes involving biosynthesis, energy supply, DNA and RNA synthesis, essential coenzymes, and regulatory mechanisms. They are synthesised in cells by *de novo* pathways, which are energy-consuming, and recycle by salvage pathways, which conserve cellular energy. While some bacteria synthesise their nucleotides *de novo*, all known microbiota are able to recycle their nucleotides. Some bacteria, including lactic acid bacteria (e.g. *Lactococcus lactis*) readily salvage external sources of purine/pyrimidine nucleosides/bases as precursors for nucleotide synthesis, to positively select for their growth in synthetic media [1,2]. Understanding the selection processes acting on the oral microbial community during human infancy may assist with development of strategies to subsequently maintain healthy gut microbiota.

Xanthine oxidase (XO) has been the focus of considerable research into ischemia reperfusion injury [3,4], however, there is increasing evidence that XO has positive physiological functions associated with the production of antibacterial reactive oxygen species (ROS) and reactive nitrogen species (RNS) [5]. XO activity has been found in the milk of all mammals studied [6], with higher activity in bovine milk than human milk [7]. XO is a major protein component of the milk fat globule membrane surrounding the fat droplets that form a suspension in freshly-expressed milk [8]. In the presence of its biochemical substrates (hypoxanthine or xanthine), XO produces microbiocidal superoxide and hydrogen peroxide (H_2_O_2_).

H_2_O_2_ subsequently acts as a substrate for the milk enzyme lactoperoxidase (LPO) present in both milk and saliva to convert–for example–dietary thiocyanate into the antibacterial ROS hypothiocyanate. The primary role of XO is thus considered to be antimicrobial, in both the milk glands (to prevent mastitis) and the milk (to reduce bacterial load) [9], acting in combination with milk LPO in milk: this is known as the 'LPO system' [10,11]. XO has also been shown to catalyse the anaerobic reduction of inorganic nitrite to nitric oxide [12,13]. Superoxide generated by XO in the presence of molecular oxygen reacts rapidly with nitric oxide to yield the RNS peroxynitrite, a powerful antibacterial agent [14,15]. Importantly, the LPO mechanism relies for its activation on the presence of XO substrates.

We discovered substantially raised concentrations of xanthine and hypoxanthine in neonatal saliva and proposed that these two substrates should react with milk XO during breast-feeding to produce H_2_O_2_. We then asked: Is the amount of H_2_O_2_ produced during breast-feeding sufficient to activate the LPO system and produce microbial inhibition? Combined with increased concentrations of growth-stimulating nucleotide precursors also found in neonatal saliva, we investigated whether there may be a unique metabolic relationship between infant and mother during the breast-feeding period to regulate oral–and hence gut–microbiota, and consequently enhance the innate immunity of the neonate.

## Results

**Fig 1:**
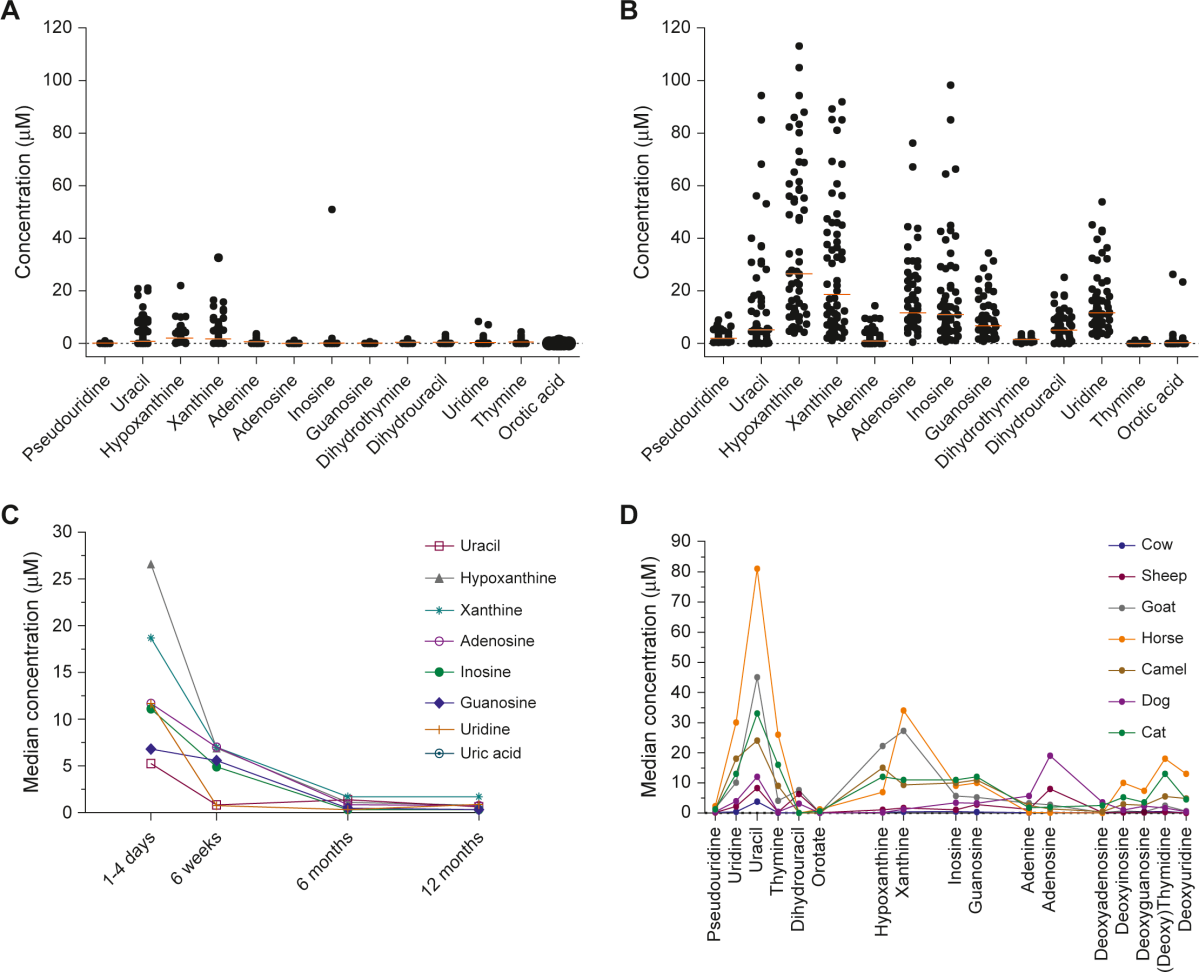
Concentration of nucleotide precursors in whole saliva samples of human adults, term neonates, and domesticated mammals. (A) Healthy non-smoking adults (n = 77) and (B) healthy full-term vaginally-delivered neonates (n = 60), lines show median values. Non-parametric analyses were conducted to estimate the significance using a Mann-Whitney U test, (C) Longitudinal study of median concentrations of metabolites in saliva from full-term neonates aged 1–4 days (n = 60), 6 weeks (n = 20), 6 months (n = 19), and 12 months (n = 14), (D) Median metabolite concentrations in saliva from selected domesticated mammals (8 cows, 5 sheep, 4 goats, 5 horses, 1 camel, 7 dogs, 5 cats). Metabolites were divided into five functional groups: Pyrimidines (Pseudouridine to Orotate); Purine bases (Hypoxanthine, Xanthine); Purine nucleosides (Inosine, Guanosine); ATP precursors (Adenine, Adenosine); Deoxynucleosides (Deoxyadenosine to Deoxyuridine).

Among 77 adults that we screened, we found low or undetectable concentrations of nucleotide metabolites (excluding urate) except in 9 adults **(Fig 1A)**. One adult had an anomalously high inosine, while eight subjects had mildly raised xanthine/hypoxanthine (the substrates of XO). The demographic parameters of 60 neonates are shown in **Table 1**. Among the neonates, median concentrations of salivary hypoxanthine and xanthine were ten-fold higher (27 μM and 19 μM respectively) than median adult values (2.1 and 1.7 μM respectively) (*p* <0.05) **(Fig 1B)**. Interestingly, while some nucleosides and bases were raised in the neonates, others such as pseudouridine, thymine and dihydrothymine were always low, while deoxy-nucleosides were undetectable. The pyrimidine base orotic acid was interesting: although the median concentrations were less than 1 μM for most adults and neonates, several neonatal saliva samples exceeded 10 μM orotic acid.

**Table 1:** Physical information of the participating neonates.

	Male	Female	Total	*p* value
***N***	22	38	60	n.s.
**Gestational age (weeks)**				
** Mean±SD**	40±1.4	40±1.3	40±1.3	0.5 (n.s.)
** Range**	38–42	37–42	37–42	
**Birth weight (g)**				
** Mean±SD**	3751±467	3665±435	3697±445	0.5 (n.s.)
** Range**	2630–4685	2768–4446	2630–4685	
**Age (hours)**				
** Mean±SD**	34±14	34±16	34±14	0.9 (n.s.)
** Range**	8–62	8–80	8–80	

To gain insights into the transitioning of these purine and pyrimidine metabolites in saliva from the high levels seen in the infants to the low levels of the adult pattern, we conducted a 12-month longitudinal study of 14 breast-fed infants. All metabolites reached the adult level after weaning, but there were differences in patterns. The purine metabolites hypoxanthine, xanthine, adenosine, inosine and guanosine gradually decreased to adult levels between 6–12 months of age, while in contrast, the pyrimidine metabolites uracil and uridine decreased sharply to adult levels by 6 weeks of age **(Fig 1C)**.

To assess whether the human salivary pattern of nucleotide metabolites was unique, we analysed the patterns of purines and pyrimidines in salivas from a selection of mature domesticated mammals. Distinctive inter-species differences emerged that far exceeded any inter-individual differences **(Fig 1D).** Details of the data are shown in **S1 Table**. Similar to humans, salivary deoxy-nucleosides were generally negligible in most species, except horse saliva, which uniquely contained raised levels of deoxy-uridine, deoxy-inosine, deoxy-thymidine and deoxy-guanosine. But in contrast to human adults, all of the adult mammals had raised levels of nucleotide metabolites, with uracil being the most prevalent metabolite, the highest concentration in horse saliva (median 80.5 μM).

**Fig 2:**
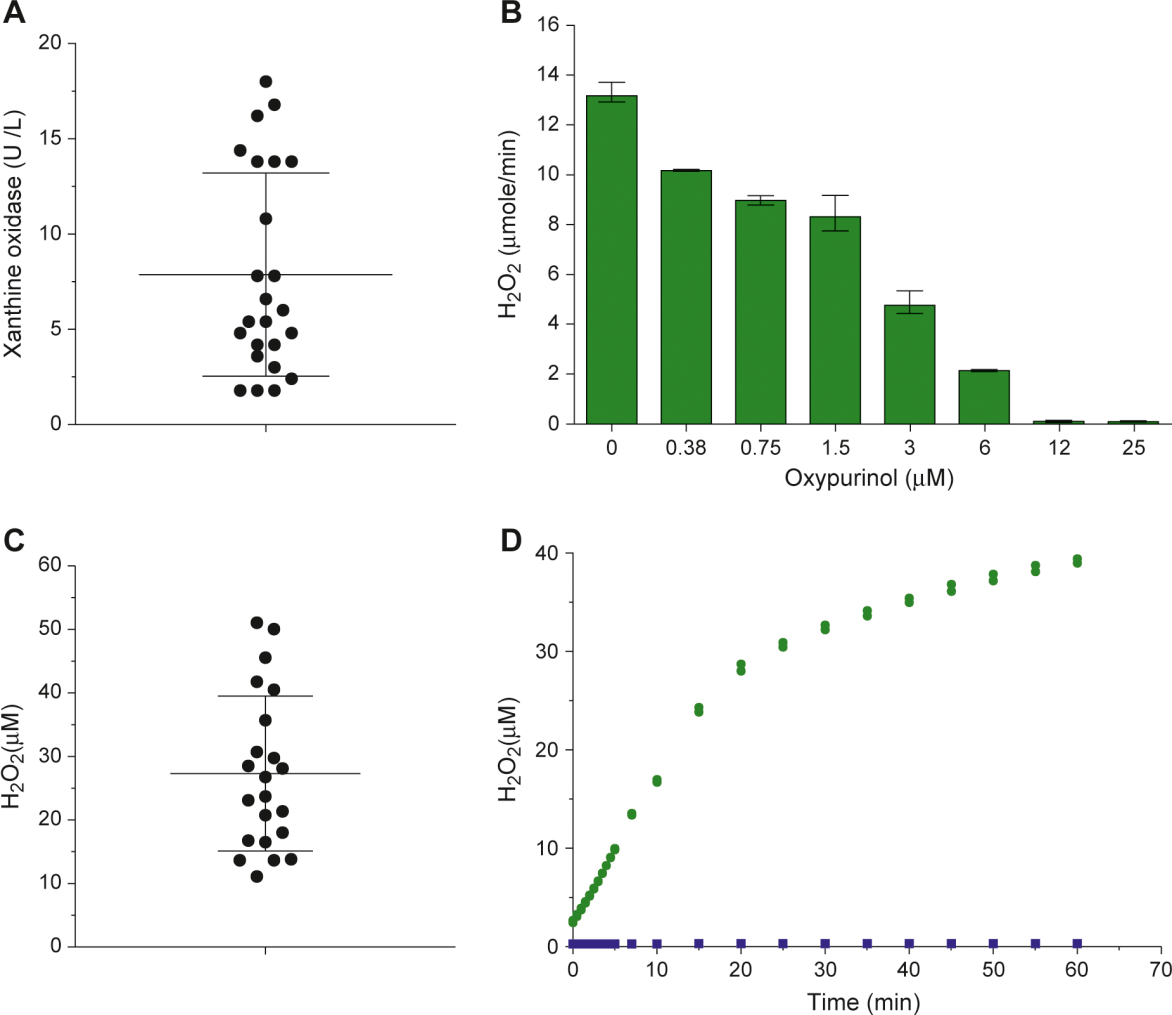
Breastmilk XO generates H_2_O_2_ when interacted with neonatal saliva. (A) Distribution of XO activity in breastmilk. Samples of colostrum were collected 1–5 day postpartum (n = 24). Error bars show mean±SD. XO activity was 8.0 ± 5.3 U/L. (B) Dose dependent inhibition of breastmilk XO by oxypurinol. Pooled breastmilk samples containing 0–25 μM oxypurinol concentrations (n = 3) were incubated with the peroxidase reagent containing 400 uM hypoxanthine. (C) The mean concentration of H_2_O_2_ in fresh breastmilk samples (n = 22) was 27.3±12.2 μM. (D) Kinetics of H_2_O_2_ generation after mixing 33 μL of diluted breastmilk, 33 μL of 1/3 diluted neonatal saliva and 34 μL peroxidase assay working solution (green circles). The negative control was buffer and peroxidase reagents (blue squares), (n = 2). The original concentrations of hypoxanthine and xanthine in the neonatal saliva were 70 μM and 30 μM respectively. Calculation of [H_2_O_2_] was based on two-fold dilutions assuming that breastmilk and neonatal saliva is 1:1 during suckling (1 mole xanthine produces 1 mole H_2_O_2_, 1 mole hypoxanthine generates 2 moles H_2_O_2_).

In view of the unexpected presence of high levels of xanthine and hypoxanthine in human neonatal saliva, we formed a hypothesis that this phenomenon must be linked to the presence of high activity XO in mammalian milk. We assayed the *in situ* XO activity–localised within the milk fat globule membranes–of 24 breastmilk samples found a mean of 8.0±5.3 U/L (apparent *K*_*m*_ = 12.8±2.1 μM, and apparent *V*_*max*_ = 8.9±6.2 μmol/min) **(Fig 2A)**. We could not detect any XO activity in pasteurised breastmilk, and not in infant dried-milk formula nor in pasteurised bovine milk, demonstrating that milk XO is inactive by commercial heat-pasteurisation. H_2_O_2_ production by breastmilk was inhibited by oxypurinol (an XO-specific inhibitor) **(Fig 2B)** in a dose-dependent manner, complete inhibition being achieved with 12 μM oxypurinol (apparent Ki = 0.6 μM), confirming that the role of XO activity in H_2_O_2_ generation.

Freshly expressed milk contains H_2_O_2_ made by XO in the milk gland lumen. We determined the mean endogenous H_2_O_2_ concentration in 24 fresh breastmilk samples to be 27.3±12.2 μM **(Fig 2C)**. When breastmilk was mixed with a neonatal saliva (containing endogenous 30 μM xanthine and 70 μM hypoxanthine), the interaction yielded an additional 40 μM H_2_O_2_ during a 1 h incubation **(Fig 2D)**.

**Fig 3:**
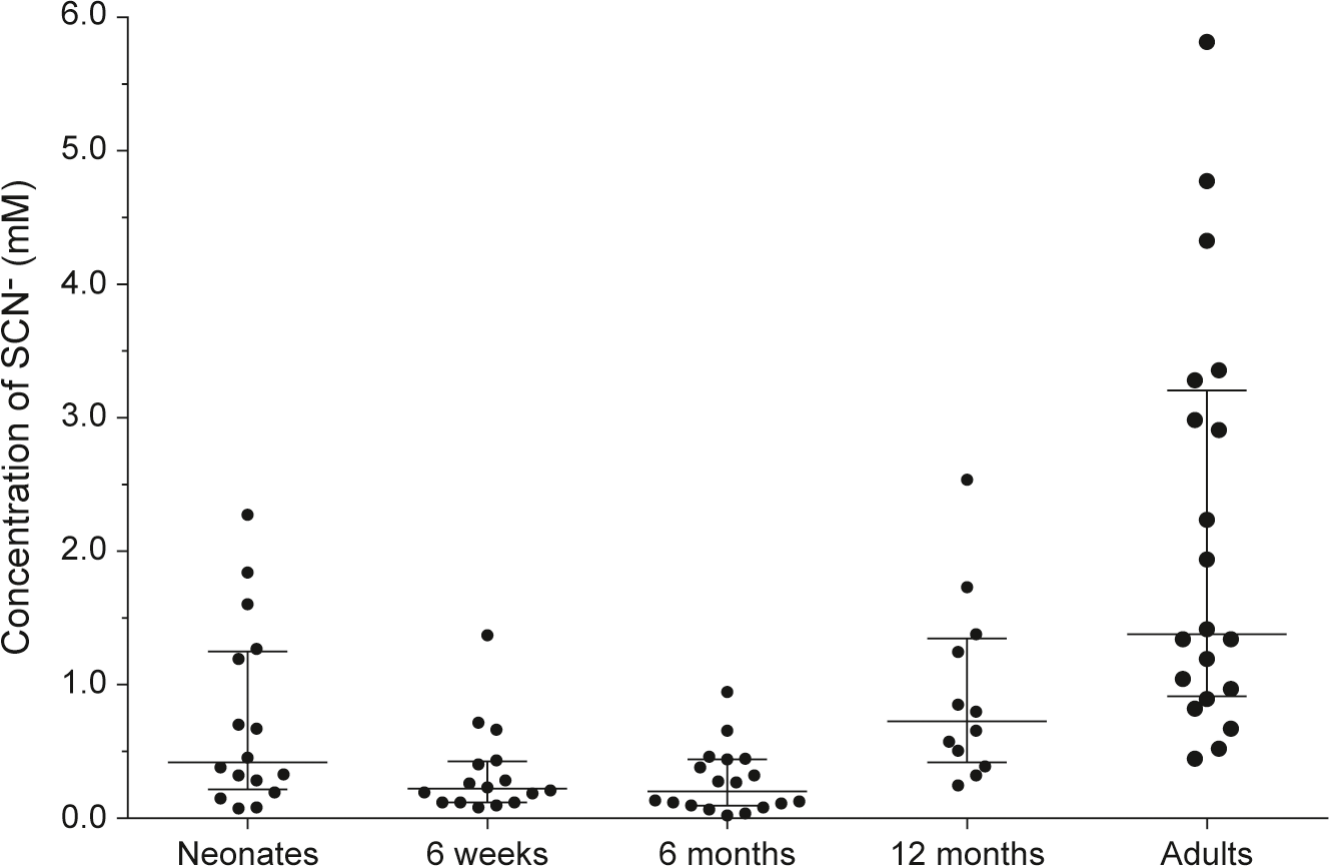
Thiocyanate in saliva. Median concentrations and interquartile ranges (mM) of thiocyanate (SCN^-^) in saliva of neonates (n = 16), infants at 6 weeks (n = 16), 6 months (n = 18), and 12 months (n = 12), and adults (n = 20).

Salivary peroxidase (SPO) is present in adult saliva, but we found that the distribution of neonatal SPO activity was highly variable and non-parametric, mostly being low to negligible with just a few samples having activity in the adult range (median 7 U/L, range 2–449, n = 12), while the distribution of SPO activity in adults was parametric (median 620 U/L, 48–1348, n = 12). Breastmilk LPO activity, which ranged from 4–90 U/L (n = 14), was relatively low compared to adult SPO but similar to neonatal SPO. Thiocyanate is considered to be an important peroxidase substrate. In neonate saliva the median concentration was 0.42 mM (with a wide range of 0.08–3.20 mM), then it declined over the weaning period before rising again to an adult median of 1.38 mM (0.45–5.82 mM) (**Fig 3**), these medians differing significantly (p = 0.0015).

**Fig 4:**
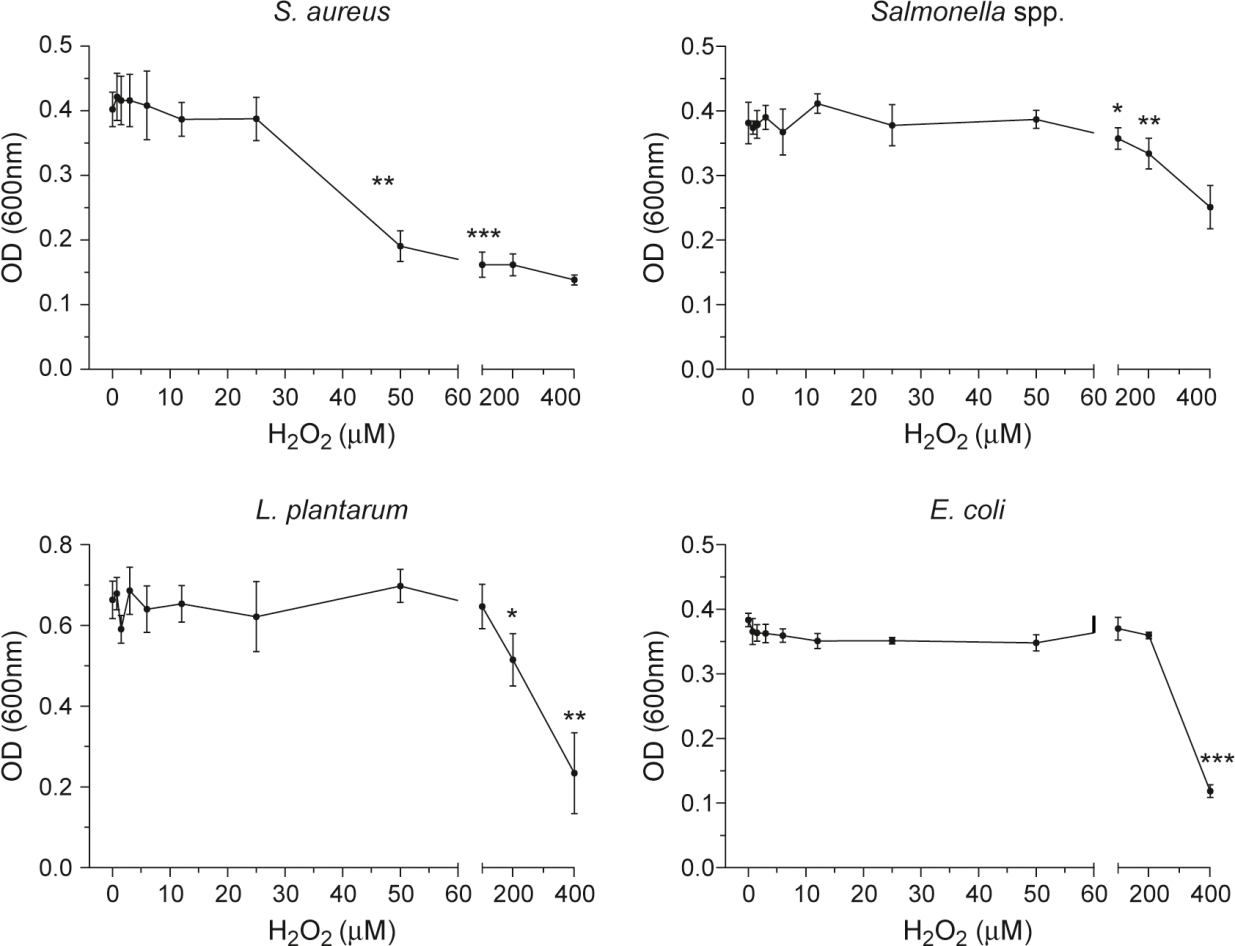
Effect of H_2_O_2_ on bacterial growth. The effect of increasing H_2_O_*2*_ concentration (0–400 μM) on bacterial growth. Hydrogen peroxide was added to nutrient broth inoculated with bacteria and incubated for 24 h at 37°C, except for *L*. *plantarum*, which was incubated for 48 h. The concentration of cells was determined as turbidity by measuring absorbance at 600 nm. Values represent the mean±SD (n = 3).

We then tested the effects on *in vitro* growth of four bacterial species by micromolar concentrations of H_2_O_2_ in standard nutrient growth media. Our results demonstrated a remarkable sensitivity of the opportunistic pathogen (Gram-positive) *Staphylococcus aureus* to H_2_O_2_ in the range of 25–100 μM, while growth of Gram-negative opportunistic pathogen *Salmonella* spp, the commensal (Gram-positive) *Lactobacillus plantarum* and gut commensal (Gram-negative) *Escherichia coli* were not affected until the H_2_O_2_ concentration exceeded 100 μM **(Fig 4)**.

**Fig 5:**
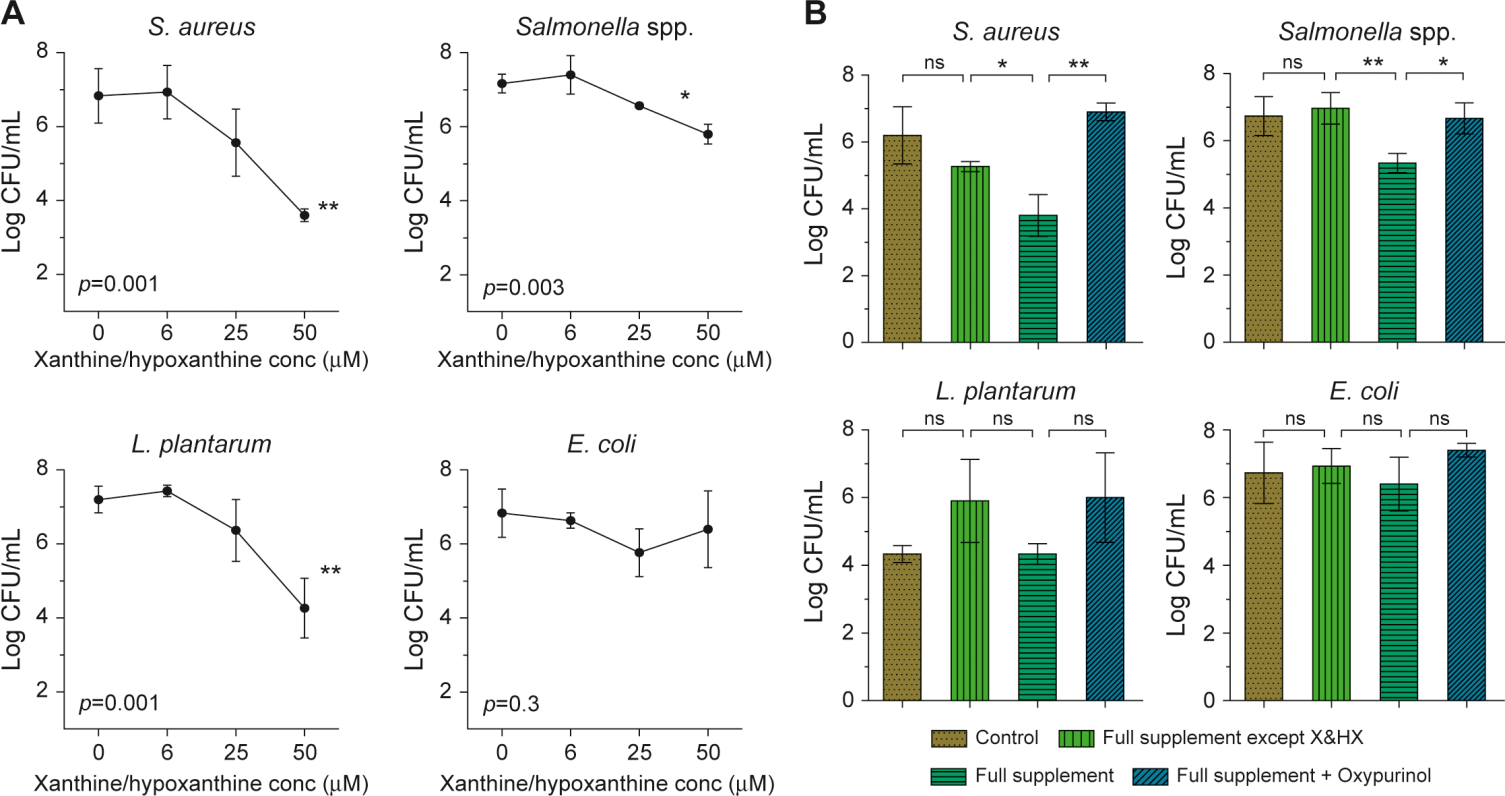
Salivary nucleotide metabolites and XO regulate bacterial growth. Bacteria were incubated with breast milk and simulated neonatal saliva for 24 h at 37°C and then enumerated by sub-culturing onto agar plates. Values represent the mean±SD (n = 3). (A) Bactericidal effects of H_2_O_2_ generated by breastmilk (XO) and increasing concentrations of salivary xanthine and hypoxanthine (0–50 μM). Figure displays the concentration-dependent effect of xanthine and hypoxanthine *versus* log CFU/mL. ANOVA was used to compare between multiple means. (B) The effect of breastmilk plus simulated neonatal saliva (with and without supplements) on bacterial counts (CFU/mL). Bacteria were incubated with breastmilk and saliva alone (Control), or supplemented with nucleotide metabolites omitting xanthine/hypoxanthine (Full supplement except X&HX), or the latter with xanthine/hypoxanthine (Full supplement), or the latter with oxypurinol (Full supplement + Oxypurinol) (see Table 2). Means were compared by t-test: * *p*<0.05; ** *p*<0.01; ns, not significant.

To demonstrate inhibition of bacterial growth under physiological conditions emulating breast-feeding, we then studied the viability of these bacteria in a medium comprising breastmilk mixed with ‘simulated neonatal saliva’ supplemented with serial dilutions of hypoxanthine and xanthine, which potentially generated 18–150 μM H_2_O_2_ and activated the milk LPO system. The breastmilk-saliva mixture inhibited, in a dose-dependent manner, the viability of *S*. *aureus*, *Salmonella* spp and *L*. *plantarum*, whereas *E*. *coli* was unaffected **(Fig 5A)**.

We also tested the effects on these four bacterial species of breastmilk and 'simulated neonatal saliva' supplemented with nucleotide metabolites, and observed marked differences in growth response between the bacteria **(Table 2)**. Supplementation of a breastmilk-saliva mixture with these purine/pyrimidine nucleosides and bases–but excluding the XO substrates hypoxanthine and xanthine–increased the count (CFU/mL) of *L*. *plantarum* by about 50%, although this increase did not reach significance, while *S*. *aureus*, *Salmonella* spp, and *E*.*coli* showed no overall increase in counts with the experimental mixture **(Fig 5B)**. When hypoxanthine and xanthine (which activate XO) were included in the metabolite mix to recreate a typical neonatal saliva profile, the counts of *S*. *aureus* and *Salmonella* spp. were significantly decreased and the improved response of *L*. *plantarum* was negated, while there continued to be no response by *E*. *coli*. When oxypurinol was added–to prevent XO generation of H_2_O_2_ –the loss of *S*. *aureus*, *Salmonella* spp. and *L*. *plantarum* was reversed.

**Table 2:** Supplemented saliva. Four 'simulated neonatal saliva' formulations were used to evaluate *in vitro* the bactericidal activity of mixtures of breast milk and simulated neonatal saliva. Saliva was collected from adults, heat-inactivated, sterilised by filtration, then supplemented with bases and/or nucleosides. The saliva concentrations of hypoxanthine (HX), xanthine (X), inosine, adenosine, guanosine, uracil and uridine were 27, 20, 11, 12, 7, 5.3 and 12 μM respectively.

Saliva	Supplements
**Control**	No supplements
**Full supplement except X&HX**	inosine, adenosine, guanosine, uracil, uridine
**Full supplement**	hypoxanthine, xanthine, inosine, adenosine, guanosine, uracil, uridine
**Full supplement + oxypurinol**	hypoxanthine, xanthine, inosine, adenosine, guanosine, uracil, uridine, plus oxypurinol (100 μM)

## Discussion

### Nucleotide Metabolites in Neonatal Saliva

In an exploration of the physiological role of neonatal salivary nucleotide precursors, we serendipitously discovered a biochemical mechanism that may be an important factor in the improved health outcomes observed with breast-feeding: maternal milk interacts with the saliva of the neonate during suckling, producing peroxide. An *in vitro* model for regulation of bacteria present in the neonate’s oral microbiota demonstrated a significant and discriminatory effect on the growth of these bacteria. Regulation of the oral microbiota of neonates is important for their subsequent health, because the gut is colonised by bacteria from the mouth. A recent review [16] reports early colonisation of the fetal gut by bacteria via swallowing *in utero*; the fetus becomes further colonised by bacteria from the placental microbiome [17]. The placental microbiome is very similar to the human oral microbiome and the full-term neonatal gut microbiome; colonised largely by Actinobacteria, Proteobacteria, Bacteroidetes and Firmicutes. Post-natally, the exposure of the infant to the mother's skin microbiota during breast-feeding also influences the gut microbiota. Thus the range of oral microbial species that the infant is exposed to orally, and any subsequent selection among those species, determines the establishment of the gut microbiome [16].

Only one group of workers have studied salivary nucleotide metabolites, reporting low concentrations (1–5 μM) of inosine, xanthine and hypoxanthine in adult saliva [18]. In plasma, nucleotide metabolite concentrations are usually less than 1 μM, with the exception of uric acid and uridine [19,20]. We confirmed these residual levels of purine/pyrimidine nucleosides and bases in adult saliva, although several adults exhibited salivary patterns that carried echoes of the neonatal pattern–a variance that may arise from genetic polymorphism in the population, for example, of nucleoside transporters. We demonstrated that the neonatal salivary pattern evolved into the adult pattern over a period between 6 weeks and 6 months, with an apparent difference between purines and pyrimidines. Such an early transition was unexpected but in retrospect this appears to confirm a link between the neonatal pattern and weaning.

We could exclude dental flora, pathological conditions and neonatal stress as the cause of raised salivary metabolite concentrations in neonates, which suggests that specific transport mechanisms may be active in the salivary glands of neonates. We have pointed out that salivary hypoxanthine and xanthine can stimulate peroxide production *via* XO, thus activating the ‘LPO system’. Other salivary nucleosides and bases can be readily utilised by the nucleotide salvage pathways in heterotrophic oral bacteria [1], or when nucleosides/bases are swallowed in saliva they can be salvaged by gut apical cells to form nucleotides: it has been reported that rapidly growing tissues including intestinal epithelium cells lack significant capacity for *de novo* synthesis of nucleotides and require exogenous sources of purine and pyrimidine bases and nucleosides [21].

In the case of the nucleobase uracil, which was grossly raised in the saliva of human neonates and the animals surveyed, mammalian cells cannot salvage this to form pyrimidine nucleotides, but uracil can be utilised by commensal bacteria [2]. Similarly, deoxy-nucleosides are generally not salvaged by mammalian cells, but uniquely horse saliva contained 10–20 μM deoxy-nucleosides, which were low or undetectable in most of the other mammals, perhaps pointing towards a specific role for uptake of deoxy-nucleosides by oral or gut bacteria in horses. This demonstrated that species-specific mechanisms for nucleotide salvage exist. These differences in saliva are echoed in milk itself where, for example, bovine milk has high concentrations of orotic acid [22] as a source of pyrimidine for suckling calves, whereas human milk does not contain orotic acid but uridine as the pyrimidine source for neonates [23]. Interestingly, the raised orotic acid that we observed in several neonatal salivas was able to be correlated with bottle-feeding of the neonates with formula (bovine) milk.

### Breastmilk Xanthine Oxidase Activity and Kinetic Parameters

Our kinetic studies of breastmilk XO *in situ* within milk fat globules found an apparent *K*_*m*_ for hypoxanthine of 12.8 ± 2.1 μM, which was comparable with published values for the purified enzyme with hypoxanthine (*K*_*m*_ = 8 μM) [24] or xanthine (*K*_*m*_ = 18 μM) [25] as substrate.

The dose-dependent inhibition of H_2_O_2_ formation in breastmilk treated with oxypurinol was consistent with its action as a specific inhibitor of XO [26], and confirmed that XO was responsible for the H_2_O_2_ production observed for the peroxidase assay. This was important for the design and interpretation of subsequent studies of bacterial inhibition by milk-saliva mixtures. Within the liver and small intestine, the major sites for xanthine oxidoreductase activity in mammals (aside from milk), the enzyme is in the form of xanthine dehydrogenase (XDH), however extracellular xanthine oxidoreductase is thought to exist solely as XO, e.g. cellular XDH is transformed to plasma XO by serum proteases while in other tissues the transformation is thought to be the result of oxidation of critical amino acids within the enzyme. Milk contains high amounts of proteases [27], and it has been suggested that these may be responsible for XO as the dominant form in milk [10].

### Absence of Xanthine Oxidase Activity in Infant Formula and Pasteurised Milk

Perhaps unsurprisingly, XO activity was undetectable in infant milk formula, indicating that the preparation, processing and manufacturing of milk formula (from bovine milk) leads to complete loss of the enzyme activity, which means that formula-fed babies do not experience oral H_2_O_2_ generation nor the LPO system.

Similarly, XO activity was not detected in pasteurised breastmilk. Pasteurised human milk has been developed specifically for babies whose mothers are unable to breast feed for medical or other reasons, as breastmilk is considered more 'natural' and possibly less allergenic than bovine milk. However, the status of XO activity in this product was previously unknown, but our findings were consistent with previous reports of significant activity loss of essential enzymes and proteins such as lysozyme [28,29], LPO, secretory IgA and lactoferrin [28] following thermal pasteurisation of breastmilk. Several studies have shown that human milk thermal pasteurisation also induced a significant loss of bactericidal capacity against *E*. *coli* [30], or both *E*. *coli* and *S*. *aureus* [31].

### Hydrogen Peroxide in Breastmilk

We found a mean H_2_O_2_ concentration of 27 μM in breastmilk, which was highly consistent with a published value of 25 μM for first-week breastmilk samples [32]. The presence of H_2_O_2_ in fresh bovine milk has been proposed to arise from the co-secretion of XO substrates xanthine and hypoxanthine into the mammary gland lumen during continuous milk production [9,10]. This continuous generation of H_2_O_2_ within the lumen is thought to be bactericidal, which may protect the breast against mastitis–most frequently caused by *S*. *aureus* [33,34]. H_2_O_2_ in breastmilk has been reported to peak in the first few weeks after birth, then declining to about 9 μM by the fourth week of infant life: this decline in H_2_O_2_ concentration postpartum [32] is coincident with the reported decrease in milk XO activity during the same period [35]. Significantly, this also coincides with the decline of salivary hypoxanthine and xanthine in the infant. In addition to direct inhibition of bacterial growth, H_2_O_2_ generated in breastmilk is also thought to serve as a substrate for the LPO expressed in human milk, producing bactericidal oxidative products [36]. This complex system may thus provide mammary glands with passive protection against invading bacteria.

Hydrogen peroxide and other ROS and RNS may also play important roles as small-molecule second messengers. It has been demonstrated that low concentrations of H_2_O_2_, in particular around 20 μM, stimulate cell viability and facilitates adhesion, migration, and wound healing in cornea cells [37] as well as enhancing the production of T-cell growth factor interleukin-2 [38]. This is consistent with a mechanism that generation of H_2_O_2_, as measured in this study, may not only contribute to the innate immunity of neonates, but may provide the appropriate concentrations for rapid cell signalling and growth of, for example, gut cells.

### Generation of Hydrogen Peroxide in the Neonatal Mouth

We demonstrated that mixing neonatal saliva (which contains high levels of xanthine and hypoxanthine), with breastmilk led to stimulation of H_2_O_2_ production. It can be speculated that a similar mechanism occurs during breast-feeding, especially as the median levels of xanthine (19 μM) and hypoxanthine (27 μM) in neonatal saliva are higher than the *K*_*m*_ values of breastmilk XO for these two substrates.

The saliva that we used for the mixing experiment contained (median concentrations of) 70 μM hypoxanthine and 30 μM xanthine, then diluted with an equal volume of milk. Given that a mole of hypoxanthine or xanthine produces 2 moles or 1 mole of peroxide respectively when reacted with XO, the theoretical concentration of H_2_O_2_ produced by two-fold diluted saliva should have been 85 μM (i.e. 70+15 μM). However, we observed only 40 μM H_2_O_2_, which demonstrated competing consumption of H_2_O_2_, presumably by SPO and milk LPO [39]. We found high variability of SPO activity in neonate saliva–a phenomenon noted previously by Gothefors and Marklund (1975) [39]–in contrast to adults who had more consistently high activity of SPO with a mean similar to that of 750 μmol/min/L (U/L) reported by Haukioja and co-workers (2004) [40]. Interestingly, the affinity (Km = 55 μM) of LPO towards H_2_O_2_ is theoretically lower [41] than glutathione peroxidase (Km = 28 μM) [42], and this may have a role in adult saliva, whereas the millimolar affinity of catalase suggests that this enzyme has no role in catabolising H_2_O_2_ in normal saliva. The biological role of SPO in adult saliva is considered to be the breakdown of H_2_O_2_ produced by dental microflora, simultaneously protecting teeth and producing antibacterial hypothiocyanate from thiocyanate; it has been demonstrated that the major form of enzymatic H_2_O_2_ consumption in normal human tracheal secretions is due to SPO [43]. In contrast, neonates have no need for protection against tooth decay, so SPO activity is generally low [39], whereas maintenance of the neonate's innate immune system and early shaping of the 'social structure' of its oral and gut microbiota [44] may be more dependent upon the milk XO-LPO system.

Compared to later stages of infancy, we found the mean concentration (400 μM) of the peroxidase substrate thiocyanate was relatively high in neonatal saliva, in agreement with a previous study [39]. The concentration of thiocyanate in adults is dependant on dietary and smoking habits, with saliva thiocyanate in non-smokers ranging between 500–2000 μM but reaching 6000 μM in cigarette smokers due to absorption of cyanide in smoke [45]. The raised thiocyanate in newborn saliva presumably derived from maternal thiocyanate carrying over into the neonate, and this then declined over 12 months. This was similar to our observation of high urate in neonatal saliva that subsequently decreased (not shown). Thiocyanate derives primarily from dietary crucifers–the cabbage/sprout family–so that the maternal plasma levels and hence neonatal saliva are dependent upon the mother's diet. It can be assumed that the infant obtains some thiocyanate from breastmilk–although this is relatively low [39]–but upon weaning, young children tend to avoid thiocyanate-rich foods because of the bitter taste, so that thiocyanate levels in infant saliva fall, until the crucifers gradually enter the diet and thiocyanate rises towards adult levels.

### Hydrogen Peroxide Effects on Bacterial Growth in Nutrient Growth Medium

H_2_O_2_ has been used for over a century to sterilise surfaces and cleanse wounds of bacterial invasion, however, the concentrations that have been typically studied and used are 3–6% v/v H_2_O_2_, or about 1–2 mol/L. Our approach was to initially examine whether micromolar concentrations of H_2_O_2_ alone–in the absence of the XO/LPO system–would inhibit or stimulate bacterial growth. We used a turbidity assay to assess *in vitro* bacterial growth in standard nutrient growth medium supplemented with micromolar concentrations of H_2_O_2_. The results demonstrated a remarkable inhibition of the Gram-positive *S*. *aureus* by 25–100 μM H_2_O_2_, while the growth of Gram-negative *Salmonella* spp., the oral Gram-positive commensal *Lactobacillus* spp. and the gut Gram-negative commensal *Escherichia coli* were not affected by H_2_O_2_ over this low range. These results were remarkably similar to those of Thomas and co-workers (1994) who reported that micromolar concentrations of H_2_O_2_ inhibited growth of oral *Streptococcus* [46].

### Bacterial Growth in Breastmilk-Saliva Mixtures

Having demonstrated direct inhibition of *S*. *aureus* growth by micromolar H_2_O_2_, we then developed a more physiological assay, where the H_2_O_2_ was generated by XO through the interaction between saliva and breastmilk *in vitro*. This system thus also included the milk/saliva LPO system, as well as endogenous thiocyanate and other ions present in saliva. It has previously been demonstrated that addition of 100 μM hypoxanthine to milk boosts production of H_2_O_2_ and nitric oxide (which can produce microbicidal peroxynitrite), completely abolishing bacterial overgrowth in milk for at least 7 days [47].

Xanthine and hypoxanthine supplementation of the saliva-milk media to activate milk XO/LPO significantly inhibited the growth of *S*. *aureus* compared to the control and nucleoside-supplemented saliva. Inhibition of XO by oxypurinol restored normal growth, demonstrating the sensitivity of *S*. *aureus* to both direct peroxide addition and the XO-LPO system. The response of *Salmonella* spp. to saliva-milk plus was similar to *S*. *aureus*, however *Salmonella* spp. required >200 μM of direct H_2_O_2_ addition to inhibit growth. This illustrated a difference between simple titration with peroxide when compared to the presence of the XO-LPO system, where other oxidative products are present. The growth of *L*. *plantarum* was noticeably inhibited by activation of the LPO system by XO substrates, with oxypurinol restoring growth to the level of the supplemented saliva. This mechanism had no effect on *E*.*coli*. Our results thus showed that the LPO system provided a negative selective mechanism for oral microbial growth, especially during breast-feeding when saliva provided hypoxanthine and xanthine as well as thiocyanate to activate the system. This is in accord with Thomas and co-workers (1994), who found that inhibition of oral *Streptococcus* growth by SPO was potentially far more effective than H_2_O_2_ alone [46]. The demonstration of this same effect on *Helicobacter pylori* is further evidence that this oral system is part of a primal mechanism for defence against pathogens and perhaps regulation of commensal bacteria [40].

We then evaluated bacterial growth stimulation by the nucleosides and bases that we found to be present in neonatal saliva (added at average concentrations and excluding xanthine/hypoxanthine). The growth of *S*. *aureus*, *Salmonella* spp., and *E*. *coli* did not benefit from supplementation when compared to the non-supplemented control, whereas *L*. *plantarum* growth was stimulated by supplemented saliva. Despite the fact that this did not reach statistical significance compared to the control, we regard this as an exciting focus for further study, because previously published methods to measure growth stimulation with nucleoside supplementation have used standard nutrient growth media that contain high (non-physiological) concentrations of purine and pyrimidine metabolites; consequently this may have confounded the results. Our method was designed to mimic the physiological conditions of a breast-feeding infant's mouth, including an intact LPO system. Some brands of milk formulae are now supplemented with 'nucleotides' or perhaps nucleotide metabolites, but there remain important differences between bovine milk and human milk, particularly in the pyrimidine composition: as cow milk provides its pyrimidine in the form of orotate whereas breastmilk does not contain orotate but provides uridine instead. Presumably these species differences have evolved from specific requirements of either the oral microbiota and/or the immature gut cells in the young mammals. Bovine milk infant formulae and breastmilk also differ in oligosaccharide content, which appears to encourage growth of certain gut microbes: thus determination of the infant microbiome becomes a delicate interplay of selective factors [48].

The presence in neonatal saliva of nucleosides is intriguing. We thus suggest that the bases and nucleosides elevated in neonatal saliva may have roles as precursors for nucleotides. These are known to promote the growth of commensal bacteria [1,2], but this may have wider implications for the development of neonatal gut cells (which take up nucleosides), and thus for the mucosal immune system [49].

It has been shown in bovine calves that the LPO system is effective in preventing ‘scours’ (diarrhoea) [50,51]. During the supportive treatment of early preterm babies in hospital neonatal intensive care units, feeding with expressed breastmilk, which has been pasteurised and lacks the LPO system, is achieved by nasogastric intubation that by-passes saliva interaction. This procedure unfortunately prevents the saliva-breastmilk effect described here, and may be an unrecognised factor contributing to the risk of necrotising enterocolitis [52]. In addition, the absence of the LPO system in infant milk formulae may be detrimental to the health of newborns by changing the selective processes that act on oral microbiota. Our studies point to an interplay of infant saliva, containing hypoxanthine and xanthine as well as growth-promoting nucleotide precursors, with the breastmilk XO-LPO system, producing positive and negative selective pressures on the early oral microbiota that will colonise the gut in infants. This appears to be a unique biochemical synergism within the milk-feeding phenomenon that defines mammals.

## Materials and Methods

### Human Saliva Samples

Study protocols received prior written approval from the Human Research Ethics Committees (HREC) of Mater Health Services (MHS) and The University of Queensland (UQ), with informed consent. The study of regulation of oral microflora by interaction of salivary metabolites with breastmilk was permitted by MHS HREC Study Reference Number 2012_01LNR. The protocol for the study of adult saliva was permitted by MHS and UQ HRECs, Study Reference Numbers 1652M and 2011000388 respectively, with written informed consent from saliva donors. The permission for the collection of infant salivas including the longitudinal study was permitted by MHS and UQ HRECs, Study Reference Numbers 1651M and 2011000387 respectively, with written informed consent being provided by the mothers for the collection of saliva from their infants. Saliva collection from infants and adults was considered to have 'minimal' risk of harm–in fact we found that none of the infants showed any distress during collection of saliva using soft cotton swabs ('cotton buds'), which required about 30 sec. Analyses of saliva samples, as well as the validation of analysis and limit of detection of salivary nucleotide metabolites using HPLC-tandem mass spectrometry (LC-MS/MS) were conducted as previously described [53]. Samples were collected from vaginally-delivered, full-term neonates identified as Caucasian. As part of ethical requirements, acceptable statistical power for the saliva studies required 53 participants: this was increased to n = 60 for neonates and n = 77 for adults. Of the 60 neonates studied, the parents of 20 of them agreed for their infants to participate in a longitudinal study involving follow-up sampling at 6 weeks (n = 20), 6 months (n = 19), and 12 months (n = 14) after the initial collection.

### Other Mammalian Saliva Samples

Mature domesticated and farm mammals were chosen. Selection was based essentially on a rationale relating to diet (which may be reflected by oral microbiota) and digestive tract: cat: obligatory carnivore; dog: omnivore; horse, cow, sheep, goat and camel: herbivores; cows, sheep and goats are ruminants; horses have pre-ruminant sacculation; camels are pseudo-ruminants.

Sampling of animal saliva/drool using soft cotton swabs did not cause any harm or discomfort or alarm to the animals, and as such the sampling was not subject to ethics application as defined by the Animal Care and Protection Act 2001 (Queensland) pertaining to agricultural/domestic animals being sampled non-intrusively by a registered veterinary surgeon (JRW). Our registered veterinary surgeon (JRW) is an academic employee of the University of Queensland, owner of the farm animals, and had permission for access. The domestic animals were owned by (JRW). Saliva was collected from unrestrained dogs and cats prior to feeding, when preparation of food and placement into bowls stimulated salivation, which was collected as drool. Sampling of herbivorous mammals was initially problematic because of contamination by vegetation. Consequently, saliva from cows was collected as they waited in bales for routine milking, during which time they do not regurgitate their cud: loading the feeding trough at the front of the bale with silage stimulated salivation, which was then collected as drool. Saliva from sheep, horses and the camel was collected using soft cotton oral swabs during scheduled regular dental examinations, vegetative contamination of horse saliva was overcome by providing them with a sand yard for one hour prior to sampling. During dental examination sheep and goats are penned, which discourages cud-chewing. In all cases, sampling of animals required less than 20 sec, and in no circumstances was an animal restrained solely for the purpose of sampling. Saliva collection was carried out in the animal’s normal day-to-day environment, with animals that were highly conditioned to the proximity of people.

Saliva was collected from 8 cows (5 of the 8 samples using cotton swabs, 3 collected from oral drool), 5 sheep (cotton swabs), 7 dogs (cotton swabs), 5 cats (cotton swabs), 5 horses (samples collected using polystyrene plastic pipettes), 4 goats (cotton swabs), and a single camel (cotton swab). The animal studies aimed solely to compare the salivary nucleotide metabolites patterns to that of human: as no data was available to model the statistical power, sample sizes were based on the practicality of obtaining saliva samples. The single sample from a camel symbolised a commonality between Saudi Arabia and Australia.

### Breastmilk

Ethical permission for the donation of breastmilk was covered by MHS HREC Study Reference Number 2012_01LNR, with written informed consent being provided by the donor mothers. Breastmilk samples were collected from 24 mothers, 1 to 5 days postpartum, by a research nurse using sterile gloves and hand expression into 50 mL sterile containers (Sarstedt Pty Ltd, Australia). Milk samples were placed on ice without delay and then transferred to the laboratory where they were subdivided into portions for the immediate assay of endogenous H_2_O_2_, or for storage at -80°C for further analyses such as XO assays. Pre-term and term 'Aptamil Gold Plus' (Nutricia Australia Pty Ltd, Australia) infant milk formula and pasteurised breastmilk were obtained for XO activity assays from the Division of Neonatology, Mater Mothers’ Hospital. Commercial pasteurised bovine milk was purchased fresh from a local retail outlet.

### Assay of Hydrogen Peroxide

All H_2_O_2_ and linked assays were incubated in flat-bottom microtiter plates (Becton Dickinson, Australia) at 37°C with shaking, in a temperature-controlled FLUOstar Omega fluorimeter (BMG Labtech, USA), until the reaction ceased. In the presence of peroxidase, H_2_O_2_ reacts with Ampliflu Red (10-acetyl-3,7-dihydroxyphenoxazine) to produce resorufin, which was measured fluorimetrically (544 nm excitation, 590 nm emission). ‘Peroxidase reagent’ comprised 100 μM Ampliflu Red (Sigma-Aldrich Pty Ltd, Australia) and 0.8 U/mL horseradish peroxidase (Sigma-Aldrich Pty Ltd, Australia) in 100 mM Tris-HCl buffer pH7.5. The H_2_O_2_ concentration was calculated from a standard curve, with calibration of the stock H_2_O_2_ concentration using absorbance at 240 nm.

To assay endogenous milk H_2_O_2_, freshly-expressed untreated breastmilk was immediately diluted 1:5 (v/v) in 100 mM Tris-HCl buffer pH 7.5. Then 50 μL diluted milk was combined in a microtiter well with 50 μL ‘peroxidase reagent’, then monitored for 30 min in the fluorimeter.

Assay of generation of H_2_O_2_ by a mixture of neonatal saliva with breastmilk, to simulate suckling, was achieved by mixing 33 μL diluted breastmilk, 33 μL neonatal saliva and 34 μL peroxidase reagent. Concentrations of hypoxanthine and xanthine in the neonatal saliva sample were 70 μM and 30 μM respectively. A negative control contained buffer and peroxidase reagent only (n = 2). Calculation of H_2_O_2_ generation was thus based on the assumption that breastmilk and neonatal saliva mix in the neonate's mouth at an approximate 1:1 ratio during suckling, and that 1 mole xanthine produces 1 mole H_2_O_2_ while 1 mole hypoxanthine generates 2 moles H_2_O_2_.

### Xanthine Oxidase and Peroxidase Enzyme Activities

For assay of milk XO activity and kinetic parameters, breastmilk (stored at -80°C) was diluted 1:30 in Tris buffer immediately before assay. Then 50 μL diluted milk was mixed with 50 μL 'peroxidase reagent'. The XO kinetic parameters were estimated for 6 breast milk samples using hypoxanthine concentrations of 0, 8, 12.5, 25, 50, 100, 200, 300, 400 μM as the final reaction concentration. XO activity of the remaining milk samples (n = 18) were assayed directly using only 400 μM hypoxanthine. For each breast milk sample, a corresponding assay substrate blank (i.e. excluding hypoxanthine) was subtracted from each data point. The production of H_2_O_2_ was monitored for 60 min in the fluorimeter, during the oxidation of hypoxanthine to uric acid. To confirm H_2_O_2_ was produced by XO activity only, the XO-specific inhibitor oxypurinol (Sigma-Aldrich Pty Ltd, Australia) was added to the above assay mix with pooled breastmilk at a range of final concentrations up to 25 μM. The lower limit for detection of activity under the conditions of assay was 0.1 U/L, a unit (U) defined as production of l μmol H_2_O_2_ per min. XO activity was also assessed in the infant dried milk formula 'Aptamil Gold Plus', pasteurised breastmilk and pasteurised bovine milk.

Peroxidase activity in milk is generally attributed to LPO, whereas 'salivary peroxidase' (SPO) activity is considered to be a mixture of myeloperoxidase and LPO [54]. We did not attempt to discern these two peroxidases. For assay of peroxidase activity in breastmilk or saliva, 50 μL breastmilk or saliva was diluted 1:15 in calcium/magnesium-free phosphate-buffered saline (PBS), were mixed with 50 μL 100 μM Ampliflu Red and 200 μM H_2_O_2_ in PBS. The reaction was monitored in the fluorimeter as for XO activity.

### Salivary Thiocyanate Assay

Assay of thiocyanate was based on a spectrophotometric method developed for human saliva using the ferric nitrate method [55], adapted for microtiter plates.

### Inhibition of Bacterial Growth by Hydrogen Peroxide

*Escherichia coli* (ATCC 2592), *Staphylococcus aureus* (ATCC 29213), *Salmonella* species (ATCC 1311) and *Lactobacillus plantarum* (UQM 297) were obtained from the microbial culture collection of the Queensland University of Technology (Brisbane, Australia). Bacterial growth inhibition assays were conducted in sterile flat-bottom 96-microtiter plates (Thermo Scientific, Australia). To test direct inhibition by H_2_O_2_, bacterial stocks were diluted in 'Oxoid nutrient broth' (Oxoid Pty Ltd, Australia) to a final concentration of 200 colony-forming units (CFU)/mL, then 50 μL aliquots were added to microtiter plate wells with serial dilutions of H_2_O_2_ in nutrient broth to final concentrations of 400, 200, 100, 50, 25, 12, 6, 3, 1.5, 0.75, 0 μM, in triplicates. A negative control contained H_2_O_2_ and nutrient broth only. Sealed microtiter plates were incubated at 37°C for 24 h or 48 h with shaking. Bacterial growth was determined turbidometrically using a microtiter plate reader (xMark, Bio-Rad Laboratories Pty. Ltd., Australia) as absorbance at 600 nm. Oxoid nutrient broth, in common with most standard media, contains significant concentrations of purines and pyrimidines: we found hypoxanthine (121 μM), xanthine (36 μM), adenosine (19 μM), inosine (148 μM), guanosine (65 μM) and uridine (87 μM) plus other trace levels of nucleotide metabolites.

### Preparation of Simulated Neonatal Saliva

As neonatal saliva was available only in small volumes, 'simulated neonatal saliva' was prepared by pooling 50 adult salivary samples, collected by passive drooling. This was then heat-inactivated at 56°C in a water bath for 35 min with mixing, to remove antibody activity present in adult saliva but which is negligible in neonatal saliva [56]. The saliva was then sterilised by vacuum filtration through a 0.45 μm filter (Grace Discovery Sciences, Australia) to remove bacteria present in adult saliva as well as particulate matter but not mucins and other proteins. This resulting 'simulated neonatal saliva' was supplemented with hypoxanthine and xanthine, and/or inosine, adenosine, guanosine, uracil and uridine (27, 20, 11, 12, 7, 5.3, 12 μM respectively) with/without 100 μM oxypurinol, to produce four different controls. These nucleoside/base concentrations corresponded to the median values for the metabolites in neonatal saliva. The 27 μM hypoxanthine plus 20 μM xanthine can theoretically produce 74 μM H_2_O_2_ (assuming XO converts each μmole of hypoxanthine and xanthine to 2 and 1 μmole H_2_O_2_ respectively).

### Inhibition or Stimulation of Bacterial Growth by Breastmilk-Saliva Mixtures

Stock bacteria species as above were diluted in sterile PBS, not nutrient broth. Breastmilk was diluted 1:6 in sterile Tris buffer. Each microtiter well contained 50 μL of four different simulated neonatal saliva formulations, 25 μL diluted breastmilk, 25 μL bacteria to give a final concentration of 200 CFU/mL bacteria in a total volume of 100 μL (in triplicate). The sealed 96-well plates were incubated 24 h at 37°C with mixing. Aliquots were then inoculated onto agar plates, incubated and viable CFU counted. Serial dilutions of xanthine and hypoxanthine were also prepared using this assay method. The final concentrations of both xanthine and hypoxanthine were 0, 6, 25 and 50 μM, which theoretically can produce 0, 18, 75 and 150 μM H_2_O_2_.

### Statistical Analyses

The nucleotide metabolite data were not normally distributed (D’Agostino and Pearson omnibus normality test), so median values were compared between two different groups using the Mann-Whitney test and between more than two groups using the Kruskal-Wallis test. The parameters of the Michaelis-Menten kinetic model, *V*_*max*_ and *K*_*m*_, for breastmilk XO were estimated using nonlinear regression analysis. All data for XO and bacterial inhibitory studies met the assumption for normality; therefore mean values were compared between two different groups using t-test and between more than two groups using one-way ANOVA. A *p*-value of 0.05 was used as the cut-off for statistical significance.

## Supporting Information

S1 Table Nucleotide precursors (median and range, μM) in saliva of a selection of domesticated mammals. (DOCX)
