# Supplementary material for: Breastmilk-Saliva Interactions Boost Innate Immunity by Regulating the Oral Microbiome in Early Infancy
Source: PLoS One. 2015 Sep 1;10(9):e0135047. doi: 10.1371/journal.pone.0135047 (PMC4556682; doi:10.1371/journal.pone.0135047)
Supplement: S1 Table — (DOCX) [file pone.0135047.s001.docx]

| Nucleotide  metabolites | Cow | | Sheep | | Goat | | Horse | | Camel | Dog | | Cat | |
| --- | --- | --- | --- | --- | --- | --- | --- | --- | --- | --- | --- | --- | --- |
|  | *(n=8)* | | *(n=5)* | | *(n=4)* | | *(n=5)* | | *(n=1)* | *(n=7)* | | *(n=5)* | |
|  | Median | Range | Median | Range | Median | Range | Median | Range | Median | Median | Range | Median | Range |
| Pseudouridine | 0.2 | ^*^<0.2-0.5 | 0.2 | 0.2-0.4 | 1.6 | 1.5-1.7 | 2.3 | 1.2-5.0 | 1.3 | 0.4 | <0.2-1.1 | 1.2 | 0.4-2.2 |
| Uracil | 3.8 | 1.6-5.8 | 8.3 | 3.4-9.7 | 45 | 30-61 | 81 | 28-130 | 24 | 12 | <1.5-37 | 33 | 25-190 |
| Hypoxanthine | 0.2 | <0.2-0.4 | 1.1 | 0.7-1.3 | 22 | 13-32 | 6.9 | 0.6-19 | 15 | 0.2 | <0.2-0.5 | 12 | 0.5-68 |
| Xanthine | <0.7 | <0.7 | 1.7 | 0.9-7.8 | 27 | 14-31 | 34 | 2.4-51 | 9.3 | 1.3 | <0.7-4.2 | 11 | 2.1-115 |
| Adenine | 0.1 | <0.1-0.6 | 1.2 | 0.1-1.6 | 3.3 | 1.1-4.2 | 0.2 | 0.1-2.5 | 2.5 | 5.7 | 0.1-19.3 | 1.8 | 0.2-38 |
| Adenosine | 0.2 | <0.03-1.2 | 8 | 2.1-11 | 2.7 | 1.4-4.0 | 0.1 | 0.1-0.2 | 1.4 | 19 | 0.8-36 | 2 | 0.7-10 |
| Deoxyadenosine | 0.1 | <0.02-0.3 | 0.2 | 0.1-2 | 0.2 | 0.1-0.6 | 0.04 | 0.02-0.1 | 0.5 | 3.6 | 0.04-18 | 2.5 | 0.4-2.7 |
| Deoxyuridine | <0.5 | <0.5 | <0.5 | <0.5 | <0.5 | <0.5 | 13 | 6.2-25 | 4.8 | 1.1 | <0.5-3.7 | 4.5 | 1.6-25 |
| Inosine | 0.7 | <0.2-3 | 1.1 | 1.0-2.0 | 5.7 | 4.8-27 | 9.4 | 5.4-27 | 10 | 3.4 | 0.4-11 | 10 | 1.8-31 |
| Guanosine | 0.3 | 0.03-3.9 | 2.8 | 1.9-6.5 | 5.2 | 2.7-30 | 10 | 6.2-40 | 10 | 3.3 | 0.5-6.0 | 12 | 2.6-33 |
| Deoxyinosine | <0.4 | <0.4-1.9 | <0.4 | <0.4 | 0.7 | 0.6-0.9 | 10 | 5.7-19 | 2.9 | 1.0 | <0.4-4.8 | 5.2 | 2.1-27 |
| Deoxyguanosine | 0.4 | 0.1-2.0 | 0.2 | 0.2-0.3 | 0.4 | 0.3-0.8 | 7.4 | 3.0-15 | 2.6 | 2.3 | 0.1-6.0 | 3.6 | 1.5-21 |
| Thymidine | 0.6 | <0.3-1.7 | 0.4 | <0.3-0.6 | 2.4 | 1.8-5.1 | 18 | 7.6-25 | 5.5 | 2.6 | <0.3-14 | 13 | 4.6-48 |
| Dihydrouracil | <3.0 | <3.0-6.5 | 6.3 | <3.0-10 | 7.6 | <3.0-16 | <3.0 | <3.0 | <3.0 | 5.0 | <3.0-6.3 | <3.0 | <3.0 |
| Uridine | 0.4 | 0.1-3.9 | 2.2 | 1.5-3.7 | 10 | 5.6-48 | 30 | 20-94 | 18 | 3.9 | 0.4-9.3 | 13 | 3.3-40 |
| Orotate | <0.5 | <0.5 | <0.5 | <0.5 | <0.5 | <0.5-1.0 | 1.2 | 0.5-1.4 | <0.5 | <0.5 | <0.5 | 1.3 | <0.5-3 |
| Urate | 35 | 26 -40 | 42 | 40-44 | 59 | 48-103 | 193 | 71-394 | 47 | 45 | 34-62 | 47 | 31-68 |
| Thymine | <1.5 | <1.5 | <1.5 | <1.5 | 4.1 | 2.2-10 | 26 | 12-48 | 9 | 2.1 | <1.5-8.4 | 16 | 12-75 |

**S1 Table. Nucleotide precursors (median and range, µM) in saliva of a selection of domesticated mammals.**
